# Benzophenone-3 and antinuclear antibodies in U.S. adolescents and adults ages 12-39 years

**DOI:** 10.3389/fimmu.2022.958527

**Published:** 2022-09-13

**Authors:** Christine G. Parks, Helen C. S. Meier, Todd A. Jusko, Jesse Wilkerson, Frederick W. Miller, Dale P. Sandler

**Affiliations:** ^1^Epidemiology Branch, National Institute of Environmental Health Sciences, National Institutes of Health, Durham, NC, United States; ^2^Population, Neurodevelopment and Genetics Program, Survey Research Center, Institute for Social Research, University of Michigan, Ann Arbor, MI, United States; ^3^Departments of Public Health Sciences, Environmental Medicine, and Pediatrics University of Rochester School of Medicine and Dentistry, Rochester, NY, United States; ^4^Social & Scientific Systems, Durham, NC, United States; ^5^Clinical Research Branch, National Institute of Environmental Health Sciences, National Institutes of Health, Durham, NC, United States

**Keywords:** cross-sectional studies (MeSH), antinuclear antibodies, oxybenzone, benzophenone-3, phenols, xenobiotics, sunscreen

## Abstract

**Background:**

Between 1988 and 2012, prevalence of antinuclear antibodies (ANA) increased in the U.S., especially in adolescents and non-Hispanic Whites. Female predominance of ANA suggests a role for hormonal factors, including xenobiotic exposures that may disrupt endocrine signaling. Benzophenone-3 (BP-3) is one such chemical with increasing exposure through sunscreen use. We investigated whether urinary BP-3 levels were related to ANA in adolescents and young adults.

**Methods:**

In a sample of 1,785 individuals ages 12-39 years in the National Health and Nutrition Examination Survey (NHANES; 2003-4, 2011-12), we examined cross-sectional associations of ANA (N=192; 3+ or 4+ at the 1:80 dilution, measured by HEp-2 immunofluorescence) with urinary BP-3, and other phenols bisphenol-A, triclosan, and parabens. Adjusted prevalence odds ratios (POR) were calculated in season-stratified models [winter (November-April) and summer (May-October)], given differences in sunscreen use and BP-3 concentrations.

**Results:**

BP-3 concentrations (detected in >98.5% of individuals) did not differ by ANA positivity in the summer (geometric mean, GM 30.6 ng/ml ANA-positive vs. 35.3 ANA-negative; GM ratio 1.15), but in winter were higher among ANA-positives (50.2 vs. 20.1 ANA-negative; GM ratio 2.50). ANA was associated with log_10_BP-3 in winter (POR 1.57; 95%CI 1.07-2.30 per unit increase) but not summer (0.94; 0.61, 1.44; interaction p=0.09). Triclosan, parabens, and bisphenol-A levels were unrelated to ANA overall or by season (ORs 0.64 to 1.33).

**Conclusions:**

The association of urinary BP-3 with ANA in the winter may reflect different exposure patterns or unmeasured confounders. Findings warrant replication in prospective studies and including past and year-round exposures.

## Introduction

Recent findings from the National Health and Nutrition Examination Survey (NHANES) suggest the prevalence of autoimmunity, as measured by antinuclear antibodies (ANA; at the 1:80 dilution using HEp-2 immunofluorescence), may be increasing in the U.S (1). In clinical settings, ANA are elevated among patients with systemic autoimmune diseases and can be detected years prior to diagnosis (1, 2). In the general population, autoimmune diseases are generally rare and risk factors for ANA are not well understood. Just as systemic autoimmune diseases occur more often in women, a female predominance of ANA suggests a role for hormonal factors, which may include xenobiotic exposures with endocrine effects (3, 4).

Growing evidence suggests that autoimmune diseases and the adaptive immune response may be impacted by exposure to endocrine disrupting chemicals, which are xenobiotics that can influence the function of endogenous hormones (3, 5). One class, the environmental phenols, includes bisphenol-A (BPA), triclosan, and benzophenone-3 (BP-3, oxybenzone). BP-3, a widely used chemical sunscreen, is rapidly absorbed through the skin, excreted in urine, and was detected in nearly 97% of the U.S. population in 2003-2004 (6, 7). Measured urinary BP-3 levels in humans have increased in the U.S. population, especially in white individuals, paralleling trends in increasing sunscreen use and incorporation of sunscreens into diverse personal care products such as moistures and cosmetics (8, 9).

We hypothesized that BP-3 could be related to higher ANA prevalence. We analyzed data from two NHANES cycles (2003-2004 and 2011-2012) with available data on both BP-3 and ANA to examine their association in adolescents and young adults (ages 12-39). We also examined associations of ANA with other phenols (BPA and triclosan), as well as parabens, another class of endocrine disruptors (measured in 2003-2004) found in sunscreen and other personal care products (9–11). Given seasonal differences in sunscreen use, associations were examined separately for the summer (May-October) and winter (November-April) NHANES samples.

## Methods

### Design and sample

The NHANES is a nationally representative sample of the non-institutionalized U.S. population, with data collected in consecutive two-year cycles since 1999. This cross-sectional study included 1941 participants ages 12-39 with existing data on ANA and urinary phenols from two survey cycles: from 2003-2004 (N=1023) and 2011-2012 (N=918). Participants completed questionnaires and most provided a blood specimen during an in-person examination. Enrollment followed a seasonal pattern such that those in more northern states were sampled in the summertime (May-October), and at lower latitudes in the winter (November-April). The NHANES protocol was approved by the Institutional Review Board of the Centers for Disease Control and Prevention (CDC). All participants provided informed consent.

### ANA assessment

The study used existing data on ANA (12). Serum samples were shipped cold and stored at -80°C, until they were tested at the 1:80 dilution using the HEp-2 indirect immunofluorescence assay NOVA Lite HEp-2 ANA slide with DAPI kit (INOVA Diagnostics, San Diego, CA), and using a highly specific fluorescein isothiocyanate (FITC)-conjugated secondary antibody (goat anti-human IgG). Images were read using the NOVA View automated fluorescence microscope system (INOVA Diagnostics). Staining intensity was rated on a scale of 0 to 4; ANA positivity was defined as any signal above zero. Assays were performed in a single laboratory and evaluated independently by two experienced reviewers who agreed on > 95% of overall ratings, with differences revolved by consensus or a third reviewer. A random sample of samples were rated with over 98% concordance.

### Phenols and parabens

Spot urine samples were collected and stored at -20°C until assays were performed. Exposures evaluated in the current study included three phenols (BP-3, BPA, triclosan; available on both cycles), and four parabens (butyl paraben, ethyl paraben, methyl paraben, and propyl paraben; available only in 2003-2004). Concentrations were measured by solid phase extraction, high performance liquid chromatography and tandem mass spectrometry as described: (https://wwwn.cdc.gov/nchs/data/nhanes/2011-2012/labmethods/EPH_G_met.pdf. Limit of detection (LOD) was 0.4 ng/mL for BP-3 and BPA, 2.3 ng/mL for triclosan, 0.2, 1.0, 1.0, and 0.2, respectively for butyl, ethyl, methyl, and propyl paraben. Samples below the LOD were assigned by dividing the LOD by the square-root of 2. Urine concentrations were adjusted indirectly by including creatinine as a covariate in multivariable models. Modest correlations were seen for methyl paraben with BP-3 and BPA in the winter sample (r=0.30 and 0.31), and among the parabens (r range 0.31-0.84).

### Covariates

Participant characteristics were collected through questionnaires and in-person visits. Covariates included age (continuous as an adjustment factor, and categorical for stratified models – ages 12-29, 20-39 years), gender (female, male), race/ethnicity (white, African American, other), education (< high school, high school grad/equivalent, some college or above), smoking, vitamin D, and body mass index (BMI). As previously described, BMI was grouped as underweight/healthy (<25 kg/m^2^), overweight (25>30 kg/m^2)^), and obese (30+ kg/m^2^) in adults 20 to 39 years and applying the 2000 CDC growth chart percentiles of <85, 85< 95, 95+ for those ages 12 to 19, and current smoking was based on cotinine concentrations as none (<0.05ng/ml), second-hand (0.05-15ng/ml) and active (>15 ng/ml) (12). Serum 25(OH)D levels were determined in 2003-2004 samples by radioimmunoassay (Diasorin, Stillwater, MN), and by liquid chromatography/tandem mass spectroscopy (LC-MS/MS) in 2011-2012 as described: (NHANES 2011-2012: Vitamin D Data Documentation, Codebook, and Frequencies (cdc.gov). Radioimmunoassay levels were harmonized to LC-MS/MS equivalents (13). Serum 25(OH)D levels grouped as: <50 nmol/L (20ng/ml, including deficient and insufficient), 50-75 nmol/L (20-30ng/ml, a debated threshold for insufficiency), and >75 nmol/L (>30 ng/ml, generally regarded as sufficiency) (13). Adults ages 20-39 were also asked about sunscreen use (“when you go out on a very sunny day for more than an hour, how often do you use sunscreen - never, rarely, sometimes, most of the time, always?”), and whether they had been diagnosed with psoriasis?

### Analysis

Analyses were performed using SAS survey procedures (version 9.4, Cary, NC, U.S.A.), and included sampling weights and design variables to account for the complex survey design of the NHANES. We first examined participant characteristics by ANA status, including demographic factors, season, BMI, smoking, cycle, and vitamin D levels; these associations with ANA were described using logistic regression models to calculate age-adjusted prevalence odds ratios (POR) and 95% confidence intervals (CI).

All analyses of BP-3 and ANA were season-specific, given prior evidence of seasonal variability in immune and inflammatory biomarkers such as total white blood cell count and C-reactive protein in adults and children in the NHANES (1999-2012) (14), sunscreen use, as well as sunlight and other unmeasured covariates, together with differential sampling of NHANES participants (i.e., Page 9: https://www.cdc.gov/nchs/data/series/sr_02/sr02_161.pdf). We first examined concentrations of BP-3 (Geometric Mean, GM, and standard deviation (GSD), by ANA status, overall and stratified by key covariates, including age, gender, race/ethnicity, vitamin D levels, and BMI. Concentrations in ANA positive versus ANA negative samples were directly compared through calculating the ratios of geometric means and 95%CI.

We estimated associations of ANA with log_10_BP-3 in multivariable logistic regression models, adjusting for creatinine, NHANES cycle, age, gender, race/ethnicity, education, current smoking, vitamin D, and smoking. In an overall model also including season, we also assessed potential multiplicative interaction to describe seasonal differences through addition of a product term (log_10_BP-3 by season); a p-value of <0.10 for the difference in -2 Log Likelihoods in the model with and without the interaction term was considered statistically significant. We also explored associations across the different demographic subgroups and vitamin D levels. Finally, we examined associations with triclosan, BPA, and parabens overall and in season-stratified models adjusting for the same model covariates except for vitamin D.

To further explore these findings, we examined the overall association by nuclear ANA pattern and staining intensity. We also conducted two sensitivity analyses for BP-3 in adults ages 20-39 years. Psoriasis is the most common autoimmune skin condition for which sunscreen might be advised; therefore, to examine potential reverse causality we excluded from the analysis individuals who reported having psoriasis who were ANA positive (4.3%) and 36 (1.7%) who were ANA-negative. We also examined associations of ANA with log_10_BP-3 by varying degrees of sunscreen use (always/usually, sometimes/rarely, and never).

## Results

### Sample characteristics

Overall, 192 (10.8%) of the study sample had detectable ANA (**Table 1**). The odds of being ANA positive were greater in females compared to males (age-adjusted POR 3.35;95%CI 2.14, 5.26), lower for those with a high school degree compared to those with at least some college (OR 0.45; 95%CI 0.25, 0.80), and among those who were overweight compared to normal or underweight (POR 0.53; 95%CI 0.37, 0.78). ANA did not vary significantly by race/ethnicity, season, smoking, vitamin D, or NHANES cycle.

**Table 1:** Characteristics and weighted proportions by ANA status in the U.S. population ages 12-39, in the NHANES sample with measured urinary phenols (benzophenone, BPA, and triclosan).

	ANA Negative	ANA Positive	
	(n=1,593, 89.2%)	(n=192, 10.8%)	
Characteristic	n (%)	n (%)	POR (95% CI)^1^
Age (Years)			
12 to 15	381 (18.1)	37 (17.6)	1.00 (REF)
16 to 19	379 (17.2)	57 (24.1)	1.44 (0.89-2.33)
20 to 24	250 (16.2)	25 (18.6)	1.18 (0.70-1.99)
25 to 29	189 (16.3)	20 (9.6)	0.61 (0.28-1.32)
30 to 34	207 (16.6)	26 (15.5)	0.96 (0.52-1.80)
35 to 39	187 (15.6)	27 (14.6)	0.97 (0.47-2.01)
Sex			
Male	818 (52.5)	57 (24.7)	1.00 (REF)
Female	775 (47.5)	135 (75.3)	3.35 (2.14-5.26)
Race/Ethnicity			
White	542 (62.1)	68 (66.0)	1.00 (REF)
Black	430 (13.3)	53 (12.0)	0.84 (0.53-1.33)
Other	621 (24.7)	71 (22.0)	0.85 (0.55-1.31)
Education^2,3^			
Some College or Above	797 (57.6)	118 (71.0)	1.00 (REF)
High School Graduate	349 (24.1)	34 (13.6)	0.45 (0.25-0.80)
Less than High School	408 (18.3)	39 (15.4)	0.67 (0.33-1.35)
Season of Blood Collection			
Summer (May-October)	833 (58.6)	94 (58.5)	1.00 (REF)
Winter (November-April)	760 (41.4)	98 (41.5)	1.01 (0.68-1.50)
Body Mass Index^3^			
Underweight/Normal	770 (48.7)	110 (61.3)	1.00 (REF)
Overweight	377 (25.4)	41 (16.8)	0.53 (0.37-0.78)
Obese	436 (25.9)	38 (21.9)	0.68 (0.42-1.11)
Smoking (cotinine)			
<LOD	305 (16.6)	51 (22.6)	1.00 (REF)
Secondhand (<15ng/mL)	962 (58.2)	120 (63.5)	0.79 (0.50-1.25)
Smoking (≥15ng/mL)	326 (25.2)	21 (13.9)	0.41 (0.13-1.25)
Vitamin D^3^			
<50 nmol/l	630 (28.2)	75 (27.2)	1.13 (0.71-1.79)
50-75 nmol/l	646 (42.6)	73 (37.1)	1.00 (REF)
≥75 nmol/l	317 (29.1)	43 (35.7)	1.43 (0.96-2.13)
NHANES cycle			
2003-2004	873 (75.3)	97 (70.4)	1.00 (REF)
2011-2012	720 (24.7)	95 (29.6)	1.30 (0.90-1.87)

LOD, limit of detection.

^1^Prevalence odds ratio (POR) and 95% confidence interval (CI) adjusted by age and NHANES sampling weights.

^2^Highest household education for those ages 12-19.

^3^Missing values on education for 2% (39) ANA negative and <1% (1) ANA positive, for BMI on 1% (10) ANA negative and 1% (3) ANA positive, and for Vitamin D on <1% (1) ANA positive individuals.

### BP-3 concentrations

In the summer sample, BP-3 concentrations were somewhat higher in ANA-positive individuals (35.3 ng/ml ANA-positive, GM 30.6ng/ml ANA-negative), but the ratio of these values was not different from the null (GM Ratio 1.15; 95%CI 0.63, 2.11)(**Table 2**). Ratios in the summer sample were elevated (>1.5) in adults, those with higher vitamin D (≥75 nmol/l), or an obese BMI, and inverse in those with lower vitamin D (<50 nmol/l: 0.48) or an overweight BMI: (0.58). In the winter sample, BP-3 concentrations were higher in ANA-positive versus ANA-negative individuals (50.2 ng/ml vs. 20.1 ng/ml; GM Ratio 2.50; 95%CI 1.26, 4.98). Differences in the GM ratios in the winter sample compared to the summer sample were typically driven by both the lower BP-3 concentrations in ANA-negative individuals, as well as the higher levels in some ANA-positive individuals. For example, in males and ANA-negative females, BP-3 concentrations in the winter sample were lower than in the summer sample, while ANA-positive females in the winter sample had higher levels than those in the summer sample. GM ratios in the winter sample were elevated in other subgroups, except in adolescents, males, or those with lower vitamin D (<50 nmol/l) or an obese BMI.

**Table 2:** Urinary Benzophenone-3 concentrations (ng/ml) by season and ANA positivity.

	Summer (May-October)					Winter (November-April)				
	ANA-neg		ANA-pos			ANA-neg		ANA-pos		
	N=833		N=94			N=760		N=98		
	n (%)	GM^1^(GSD)	n (%)	GM^1^ (GSD)	GM Ratio (95% CI)^2^	n (%)	GM^1^(GSD)	n (%)	GM^1^(GSD)	GM Ratio (95% CI)^2^
Total sample	833 (100)	30.6 (7.1)	94 (100)	35.3 (6.5)	1.15 (0.63-2.11)	760 (100)	20.1 (6.6)	107 (100)	50.2 (8.2)	2.50 (1.26-4.98)
Age (years)										
Adolescents (12-19)	399 (37)	29.5 (6.5)	49 (48)	29.7 (7.0)	1.01 (0.56-1.80)	361 (33)	19.1 (5.9)	45 (33)	24.2 (3.8)	1.21 (0.86-1.87)
Adults (20-39)	434 (63)	31.3 (7.4)	45 (52)	41.3 (6.0)	1.58 (0.51-3.39)	399 (67)	20.6 (7.0)	53 (67)	72.5 (10.1)	3.52 (1.41-8.79)
Gender										
Males	416 (51)	25.3 (6.8)	29 (23)	24.2 (3.6)	0.96 (0.58-1.58)	402 (55)	14.0 (5.9)	28 (28)	15.5 (4.1)	1.10 (0.60-2.02)
Females	417 (49)	37.3 (7.2)	65 (77)	39.5 (7.4)	1.06 (0.49-2.28)	358 (45)	31.3 (6.9)	70 (72)	78.7 (8.6)	2.52 (1.02-6.21)
Race/ethnicity										
White	338 (70)	36.7 (7.6)	45 (77)	41.9 (6.6)	1.14 (0.53-2.48)	204 (51)	28.7 (7.2)	23 (51)	79.9 (7.5)	2.78 (0.80-9.66)
African American	276 (15)	15.9 (4.3)	32 (13)	12.7 (5.5)	0.80 (0.44-1.45)	154 (10)	11.7 (4.8)	21 (10)	31.4 (5.5)	2.53 (1.33-5.39)
Other	219 (15)	25.8 (6.8)	17 (10)	34.5 (4.9)	1.34 (0.44-4.03)	402 (39)	14.5 (5.7)	54 (39)	31.7 (8.9)	2.18 (0.91-5.21)
Vitamin D (nmol/l)^2^										
<50 nmol/l	286 (21)	16.0 (6.7)	30 (25)	7.7 (7.1)	0.48 (0.13-1.79)	344 (39)	12.4 (6.3)	45 (30)	18.0 (5.3)	1.45 (0.67-3.10)
50-75 nmol/l	332 (42)	25.6 (6.5)	32 (28)	34.0 (4.9)	1.33 (0.52-3.39)	314 (44)	19.8 (5.4)	41 (50)	52.1 (8.6)	2.63 (1.08-6.42)
≥75 nmol/l	215 (38)	53.2 (6.9)	31 (47)	83.1 (3.9)	1.56 (0.64-3.79)	102 (17)	62.7 (7.2)	12 (19)	234 (4.9)	3.73 (1.05-13.3)
BMI^3^										
Normal/underweight	399 (49)	31.6 (8.0)	58 (60)	38.9 (6.1)	1.23 (0.77-1.97)	371 (48)	25.1 (6.9)	52 (63)	70.8 (9.0)	2.82 (0.99-8.01)
Overweight	196 (24)	32.9 (6.4)	16 (16)	19.0 (8.8)	0.58 (0.05-6.59)	181 (28)	18.0 (7.6)	25 (18)	45.1 (7.7)	2.51 (0.98-6.39)
Obese	232 (27)	27.2 (6.1)	19 (25)	41.9 (5.9)	1.54 (0.55-4.34)	204 (24)	14.6 (4.6)	19 (18)	17.5 (3.6)	1.19 (0.61-2.35)

^1^Unadjusted geometric mean (GM) and standard deviation (GSD) and GM Ratio plus 95% confidence intervals (CI) including: 16 samples with concentrations (8 ANA negative and 1 ANA positive in the summer sample and 6 ANA negative and 1 ANA positive in the winter sample) below the limit of detection (LOD) that were assigned to LOD/sqrt (2).

^2^All p-values exceed 0.05 except for values in the winter sample as follow: overall (p=0.01), adults ages 20-39 (p=0.01), females (p=0.04), African Americans (p=0.01), and those with Vitamin D of 50-74 nmol/l (p=0.03) or ≥75 nmol/l (p=0.04).

^3^Missing values on BMI on 1% (10) ANA negative and 1% (3) ANA positive, and for Vitamin D on <1% (1) ANA positive individuals.

### Multivariable analyses

**Figure 1:**
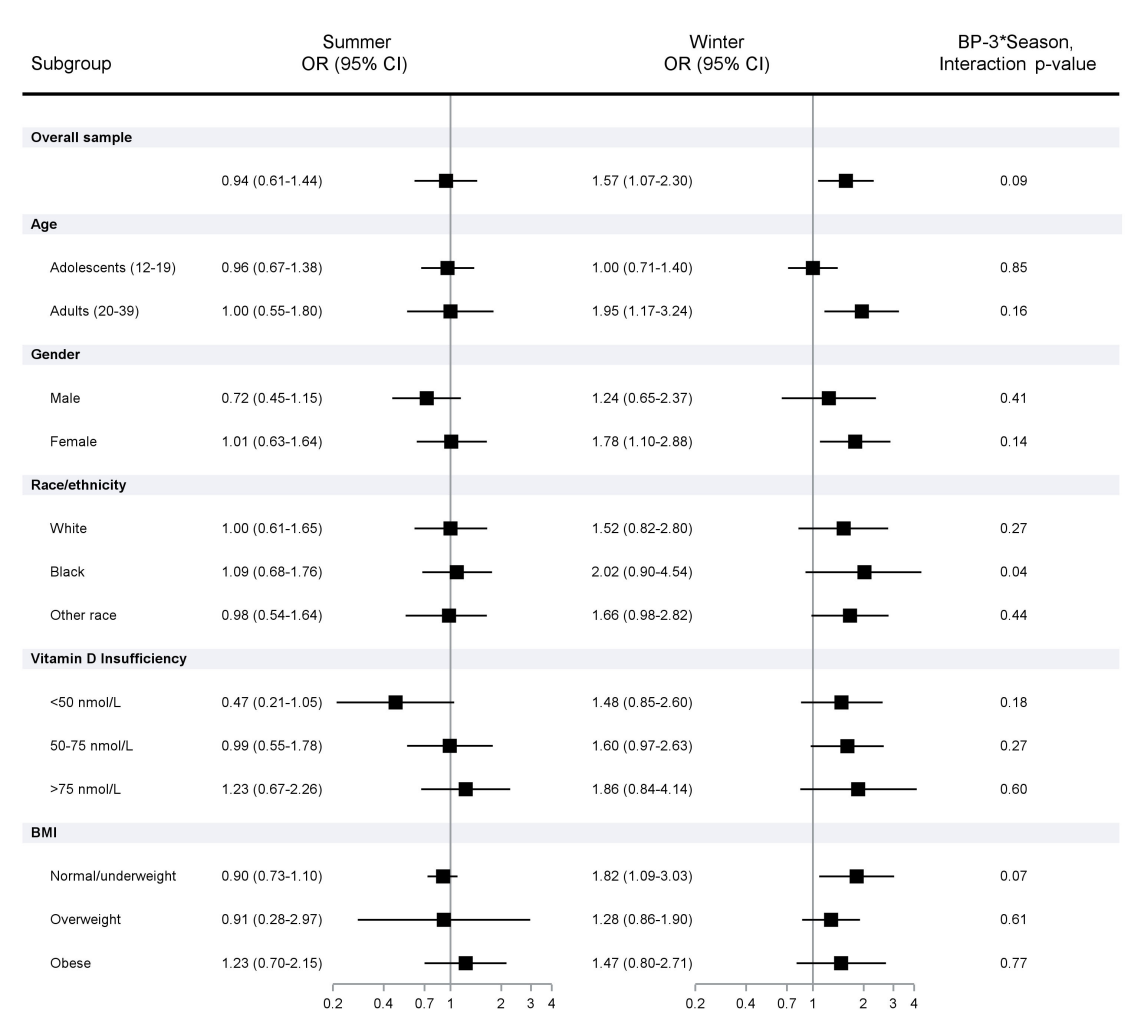
Association of ANA positivity per unit increase in urinary log_10_ BP-3 concentrations adjusted for creatine and covariates. Prevalence odds ratios and 95% Confidence Intervals calculated using logistic regression models, adjusting for urinary creatinine, NHANES cycle, age, sex, race/ethnicity, BMI, smoking (cotinine), education, and Vitamin D.

**Figure 1** shows the multivariable adjusted odds of ANA-positivity per unit increase in urinary log_10_BP-3. Higher BP-3 concentrations were associated with ANA in the winter (POR 1.57; 95%CI 1.07, 2.30) but not in the summer sample (POR 0.94; 95%CI 0.61, 1.44; interaction _int_p=0.09). No elevated PORs were seen in the summer sample subgroups, but an inverse association (POR 0.47; 95%CI 0.21, 1.05) was seen among those with vitamin D <50 nmol/l. Associations in the winter sample were most apparent among adults (POR 1.95; 95%CI 1.17, 3.24), females (POR 1.78; 95%CI 1.10, 2.88), and those with normal/underweight BMI (POR 1.82; 95%CI 1.09, 3.03). The interaction by season was especially apparent among African Americans (_int_p=0.04) and those with normal/underweight BMI (_int_p=0.07).

**Table 3** shows that the association with BP-3 in the winter sample was most apparent for those with the dense fine speckle pattern (34% of total ANA positive individuals (POR 2.37; 95%CI 1.22, 2.08) and those with a lower staining intensity (85% of ANA positives at 1-2; POR 1.61; 95%CI 1.08, 2.39). The fine speckle pattern was inversely associated with BP-3 concentrations in the summer sample. In adults ages 20-39 with available data, a similar overall association was seen in the winter sample after excluding those with psoriasis (**Table 4**). Further, a stronger association was seen in the winter among those who usually or always used sunscreen (OR 2.85; 95%CI 1.43-5.69), while an inverse association was seen among those in the summer who never used sunscreen (OR 0.48; 95%CI 0.25, 0.89; **Table 5**).

**Table 3:** Patterns and intensity of ANA and associations with BP-3, by sample season.

	ANA positive(N=192)	Summer	Winter	Interaction season X BP-3
	N (%)	Odds Ratio (95% Confidence Limit)^1^		p-val
Nuclear pattern				
Fine speckled	62 (23.3)	0.62 (0.42-0.92)	1.33 (0.86-2.08)	0.34
Dense fine speckled	58 (33.7)	1.33 (0.73-2.40)	2.37 (1.22-4.60)	0.10
Intensity level				
1-2	162 (86.3)	0.88 (0.54-1.41)	1.61 (1.08-2.39)	0.07
3-4	30 (13.7)	1.21 (0.55-2.68)	0.98 (0.56-1.73)	0.75

^1^Models of BP-3 association with ANA positivity of specific patterns or intensity compared to ANA negatives, adjusting for NHANES cycle, age, sex, race/ethnicity, creatinine, BMI, current smoking, education, and Vitamin D.

**Table 4:** Association of log_10_BP-3 with ANA, ages 20-39: excluding participants with self-reported psoriasis.

	Summer(May-October)	Winter (November-April)	Interaction
	POR (95% CI)^1^	POR (95% CI)^1^	p-value season X BP-3
Total sample	1.00 (0.55-1.80)	1.95 (1.17-3.25)	0.16
Exclude psoriasis^2^	1.00 (0.54-1.85)	1.96 (1.14-3.38)	0.21

^1^Adjusted odds ratios (OR) and 95% confidence intervals (CI) calculated using logistic regression models, adjusting for creatinine, NHANES cycle, age, sex, race/ethnicity, BMI, smoking (cotinine), education, and Vitamin D.

^2^Excluding 23 self-reported participants with psoriasis (5 ANA-pos, 18 ANA-neg).

**Table 5:** Association of log_10_BP-3 with ANA, ages 20-39, stratified by sunscreen use.

	Summer(May-November)		Winter(October-April)	
	N+/N-^1^	POR (95% CI)^2^	N+/N-^1^	POR (95% CI)^2^
Sunscreen Use				
Always/Most of the time	20/93	1.14 (0.42-3.08)	16/91	2.85 (1.43-5.69)
Sometimes/rarely	12/137	0.87 (0.42-1.82)	20/137	1.04 (0.51-2.14)
Never	11/196	0.48 (0.25-0.89)	16/158	1.61 (0.75-3.46)

^1^N+/N- = Number of ANA-positive (cases)/Number of ANA-negative (non-cases).

^2^Models adjusted for survey cycle, age, gender, race/ethnicity, creatinine, BMI, smoking status, educational attainment, and Vitamin D.

In other analyses, ANA did not appear to be associated with the phenols, triclosan and BPA, overall or in either of the two seasonal samples, nor were associations seen in the smaller subsample for the parabens; ORs ranged from 0.64 (0.31, 1.27) for methyl paraben in the winter sample to 1.33 (0.73, 2.41) for methyl paraben in the summer (**Table 6**).

**Table 6:** Association of ANA with log_10_ triclosan, BPA, and paraben concentrations overall and by season.

		Odds Ratio and (95% Confidence Limits)^1^					
	N (%)< LOD	Total sample	p-val	Summer	p-val	Winter	p-val
*Phenols*	N=1,728						
Triclosan	381 (20)	0.84 (0.65-1.09)	0.19	0.85 (0.59-1.21)	0.38	0.76 (0.49-1.19)	0.23
BPA	72 (2.6)	1.00 (0.46-2.18)	0.99	0.92 (0.30-2.85)	0.89	1.07 (0.42-2.74)	0.90
*Parabens*	N=789						
Butyl paraben	558 (71)	0.87 (0.45-1.66)	0.69	1.02 (0.32-3.22)	0.98	0.79 (0.49-1.27)	0.34
Ethyl paraben	434 (57)	1.13 (0.79-1.63)	0.52	1.16 (0.58-2.31)	0.69	1.17 (0.70-1.96)	0.56
Methyl paraben	10 (1.4)	0.85 (0.52-1.40)	0.53	1.33 (0.73-2.41)	0.36	0.64 (0.31-1.32)	0.23
Propyl paraben	33 (5.0)	0.92 (0.71-1.19)	0.54	1.02 (0.63-1.67)	0.94	0.89 (0.60-1.31)	0.57

^1^Logistic regression models adjusted for BP3, NHANES cycle, age, sex, race/ethnicity, creatinine, BMI, current smoking, education, and Vitamin D. Those with measures below the limit of detection (LOD) were replaced by LOD/sqrt (2).

## Discussion

Exposure to benzophenone-3, an ingredient in chemical sunscreens for over 40 years (15), is widespread and increased in the U.S. population at the same time as rising ANA prevalence (8, 9, 12). In this cross-sectional study of NHANES participants ages 12-39, we found that elevated urinary BP-3 concentrations were related to ANA among those who were sampled in winter, but not summer. These findings were robust to confounder adjustment and covariate stratification, providing some support for the idea that BP-3 exposure may play a role in the observed increases in ANA.

Our analyses were stratified based on the *a priori* expectation of seasonal differences in exposure patterns and circumstances of individuals sampled in the warmer months, such as greater episodic sun exposure and sunscreen use. Indeed, BP-3 concentrations were elevated among the majority of individuals sampled in summer. While season was not associated with ANA, it significantly modified the association of BP-3 with ANA. In other NHANES adult samples from 2003-2006 and 2009-2012, BP-3 concentrations were associated with more frequent sunscreen use in both the summer and winter samples (16). Interestingly, in our sensitivity analyses the association of ANA with BP-3 in the winter sample was primarily among those using sunscreen most of the time or always. This finding was based on only 16 ANA-positive individuals, however, and caution is warranted in making comparisons, especially with those in the summer with less frequent or no sunscreen use (12 and 11 ANA-positive individuals).

In the NHANES, season determines the geographic location of sampling, which may impact opportunities for longer duration and more intense sun exposure – increasing both cumulative sunscreen use and exposure to ultraviolet (UV) radiation. Notably, UV light can suppress immunity, while at the same time periodic intense exposures (and greater damage) may stimulate existing ANA production through greater antigen exposure (17, 18). Personal sun exposure and sunburn have been associated with the development of some autoimmune diseases, including lupus erythematosus (SLE) and dermatomyositis (19, 20), while in other studies UV has appeared protective for multiple sclerosis and rheumatoid arthritis (21, 22). Further investigation is needed in a larger sample to evaluate the role of UV exposure, sunburn, and sunscreen use, in relation to ANA.

Vitamin D levels may also be influenced by UV exposure (23). Lower vitamin D levels were previously found to be associated with ANA in middle aged and older adults in the NHANES (2001-2004) (24), but in the current study sample of younger adults and adolescents, higher vitamin D appeared positively associated with ANA (age-adjusted OR 1.43 for ≥75 versus 50-75 nmol/L). We saw no evidence of confounding by vitamin D levels; though in the summer sample the association of log_10_BP-3 with ANA appeared to be inverse, this may be due to chance or confounding or interactions with other unmeasured factors.

Females had higher ANA prevalence compared to males, and the association of ANA with BP-3 in this winter sample was also more apparent in females. This was largely driven by the higher BP-3 concentrations in the winter sample among ANA-positive females and may reflect underlying differences in exposure sources and patterns. In a consumer survey from 2013, nearly 43% of females regularly used sunscreen on their face, compared with 18% of males (25); regular use of sunscreen-containing cosmetics may extend year-round and lead to increased cumulative or chronic exposures. Lifelong patterns of sunscreen and personal care product use may start in adolescence, at younger ages parental influences play a role – as evidenced by the strong correlation of maternal levels with their children’s ages 6-11 in a study of 145 unique pairs (26). Interestingly, BP-3 concentrations were similar among ANA-negative adolescents and adults, both in the summer and winter samples; and the elevated BP-3 levels among ANA-positive adults in the winter sample could reflect a similar scenario as suggested for females if this reflects individuals who habitually use sunscreen year round or at an even greater frequency in the summer.

We lacked data on systemic autoimmune diseases, however, these are still relatively rare and unlikely to account for the observed differences; moreover, we saw no evidence of potential reverse causality after excluding individuals reporting psoriasis. In NHANES adults, sunscreen use was previously associated with both BP-3 and triclosan levels (9). But in the current study sample, the other phenols and parabens were neither strongly correlated with BP-3, nor were they associated with ANA overall or by season. While the finding for BP-3 appeared fairly specific, we cannot rule out the role of unmeasured confounders. We did not consider mixtures or other chemicals included in personal care products, such as phthalates, or other chemical sunscreens, some of which may also impact the immune system (27, 28).

The relationship of BP-3 with ANA could be mediated by hormone-disrupting effects (e.g., estrogenic, androgenic) either directly or through its metabolites (29–31). Experimental studies of other phenols (e.g., BPA), suggest potential effects on the development of T-cells, including regulatory T-cells. Other non-endocrine mediated effects may include BP-3 phototoxicity, or through other pathways (e.g., retinoid-X receptor) (32, 33). Research in susceptible animal models may be helpful in understanding the potential effects of BP-3 on ANA development.

Limitations of the current study include the cross-sectional design and small sample size. Our findings of seasonal differences suggest that a single measured BP-3 concentrations in spot-urines may be insufficient for capturing the relevant exposure or timing relative to the development of ANA. Similarly, urinary BPA levels in spot urines are also insufficient measures for daily or longer-term exposure, thereby introducing exposure misclassification and likely obscuring potential associations (34). The use of sunscreen containing BP-3 has been shown to lead to short-term rise in plasma levels that remain above background at 7 days (6), and while concentrations in spot urines will certainly detect recent use, levels may also be compounded among individuals routinely using other BP-3 containing products, e.g., daily moisturizers or cosmetics. BP-3 has also been found in adipose tissue along with other phenols and some parabens, but the implications of accumulation and elevated body burden have not been explored (35). We saw no evidence of confounding by BMI; however, the seasonal difference was most apparent among those who were not overweight or obese. This could be due to differences in habitual uses of sunscreen (e.g., for physical activity outdoors or sunbathing), confounders, or differential processing or excretion patterns associated with elevated body fat.

The current study sample was limited to adolescents and young adults who lived the majority of their lives following approval of BP-3 containing sunscreens in the early 1980s and were likely to be exposed at younger ages. NHANES sampling is representative U.S. population, however our findings may not be generalizable to other ages and populations. The association of BP-3 with ANA was more apparent in adults ages 20-39 and females, who may be a key demographic for use of sunscreens and sunscreen-containing cosmetics (often used year-round). We lacked data on disease-specific antibodies but found that the association with BP-3 was stronger for the nuclear dense fine speckled phenotype, typically seen in healthy populations and patients with inflammatory conditions (but not autoimmune diseases) (36, 37). We noted an unexpected inverse association in the summer sample among those with the fine speckle pattern, which is seen in some systemic autoimmune diseases (38). Interestingly, we also saw an inverse association in the summer sample among those with lower vitamin D (<50 nmol/l) or who never used sunscreen; reasons for a protective association in the summer are unclear, and the numbers are too small to disentangle these findings. While the determinants and natural history of ANA in the general population are not well understood, once immune tolerance is broken the ability to produce ANA is likely to be maintained over time, though levels may wax and wane. As ANA can be present many years prior to the development of autoimmune diseases, the longer-term implications of ANA associations and potential role of BP-3 exposure at younger ages warrant further investigation.

In conclusion, our findings partially support the idea that BP-3 concentrations may be associated with ANA in the winter NHANES sample. Given the widespread use of BP-3 containing sunscreens and other products, further research is warranted in longitudinal studies following individuals with data collected across the seasons, and exposure assessment including internal measures, but also questionnaire data on past and present, along with co-exposures, other sunscreen active ingredients and diverse exposure sources (39). In particular, given the general lack of association seen in the summer sample, better understanding is needed on the role of personal sun exposures and potential pathways through which UV could mitigate the effects of BP-3 on ANA.

## Data availability statement

Publicly available datasets were analyzed in this study. This data can be found here: Centers for Disease Control and Prevention (CDC) National Health and Nutrition Examination Survey (NHANES), https://wwwn.cdc.gov/nchs/nhanes/Default.aspx

## Ethics statement

The studies involving human participants were reviewed and approved by the Institutional Review Board of the Centers for Disease Control and Prevention (CDC). Written informed consent to participate in this study was provided by the participants.

## Author contributions

FM, DS, HM, and CP contributed to the conception and design of the study. JW performed the statistical analyses. CP, HM, TJ, and DS contributed to the design of analyses and interpretation of results. CP wrote the first draft of the manuscript. All authors helped to revise the manuscript, read, and approved the final draft for submission. All authors contributed to the article and approved the submitted version.

## Funding

This research was supported in part by the Intramural Research Program of the NIH, National Institute of Environmental Health Sciences (Z01-ES049028) and contract HHSN273201600011C to Social & Scientific Systems. HM was supported in part by the Shaw Scientist Award from the Greater Milwaukee Foundation.

## Conflict of interest

The authors declare that the research was conducted in the absence of any commercial or financial relationships that could be construed as a potential conflict of interest.

## Publisher’s note

All claims expressed in this article are solely those of the authors and do not necessarily represent those of their affiliated organizations, or those of the publisher, the editors and the reviewers. Any product that may be evaluated in this article, or claim that may be made by its manufacturer, is not guaranteed or endorsed by the publisher.
